# Designing and Validating a Survey for National-Level Data During the COVID-19 Pandemic in Sri Lanka: Cross-Sectional Mobile Phone Surveys

**DOI:** 10.2196/49708

**Published:** 2024-11-08

**Authors:** Rachael Phadnis, Udara Perera, Veronica Lea, Stacy Davlin, Juliette Lee, Casey Siesel, Dhanushka Abeygunathilaka, S C Wickramasinghe

**Affiliations:** 1 Centers for Disease Control and Prevention Foundation Atlanta, GA United States; 2 Non-Communicable Diseases Bureau, Ministry of Health Colombo Sri Lanka; 3 Centers for Disease Control and Prevention Atlanta, GA United States

**Keywords:** pilot study, mobile phone survey, survey methodology, COVID-19, data collection, national survey, pandemic, population-based study, Sri Lanka, middle-income countries, low-income countries, vaccine acceptability, vaccine, COVID-19 vaccination

## Abstract

**Background:**

The COVID-19 pandemic has generated a demand for timely data, resulting in a surge of mobile phone surveys for tracking the impacts of and responses to the pandemic. Mobile phone surveys have become a preferred mode of data collection across low- and middle-income countries.

**Objective:**

This study piloted 2 population-based, cross-sectional mobile phone surveys among Sri Lankan residents in 2020 and 2021 during the COVID-19 pandemic. The surveys aimed to gather data on knowledge, attitudes, and practices, vaccine acceptability, availability, and barriers to COVID-19 testing, and use of a medicine distribution service.

**Methods:**

The study used Surveda, an open-source survey tool developed by the NCD (noncommunicable disease) Mobile Phone Survey Data 4 Health Initiative, for data collection and management. The surveys were conducted through interactive voice response using automated, prerecorded messages in Sinhala, Tamil, and English. The sample design involved random sampling of mobile phone numbers, stratified by sex, proportional to the general population. Eligibility criteria varied between surveys, targeting adults aged 35 years and older with any noncommunicable disease for the first survey and all adults for the second survey. The data were adjusted to population estimates, and statistical analysis was conducted using SAS (SAS Institute) and R software (R Core Team). Descriptive statistics, Rao-Scott chi-square tests, and *z* tests were used to analyze the data. Response rates, cooperation rates, and productivity of the sampling approach were calculated.

**Results:**

In the first survey, n=5001, the overall response rate was 7.5%, with a completion rate of 85.6%. In the second survey, n=1250, the overall response rate was 10.9%, with a completion rate of 61.9%. Approximately 3 out of 4 adults reported that they avoided public places (888/1175, 75.6%), more than two-thirds avoided public transportation (808/1173, 68.9%), and 9 out of 10 practiced physical distancing (1046/1167, 89.7%). Approximately 1 out of 10 Sri Lankan persons reported being tested for COVID-19, and the majority of those received a polymerase chain reaction test (112/161, 70%). Significantly more males than females reported being tested for COVID-19 (98/554, 17.8% vs 61/578, 10.6%, respectively; *P*<.001). Finally, the majority of adult Sri Lankan people reported that they definitely or probably would get the COVID-19 vaccination (781/1190, 65.7%).

**Conclusions:**

The surveys revealed that, overall, the adult Sri Lankan population adhered to COVID-19 mitigation strategies. These findings underscore the use of mobile phone surveys in swiftly and easily providing essential data to inform a country’s response during the COVID-19 pandemic, obviating the need for face-to-face data collection.

## Introduction

The COVID-19 pandemic caused by the novel SARS-CoV-2 has created an unprecedented challenge for governments, public health agencies, and populations globally [1,2]. In order to mitigate the effects of COVID-19 pandemic, governments and public health agencies implemented control strategies that focused on nonpharmaceutical interventions (NPIs), which rely on reducing the contact between infected and uninfected individuals by implementing shelter-in-place, stay-at-home orders or lockdowns, travel restrictions, restrictions on social gatherings, closures of schools and businesses, and increased testing and contact tracing [3-6]. These types of interventions are effective when they result in large-scale human behavioral changes that reduce disease transmission but are challenging to maintain. It is essential to assess adherence to NPIs to fully inform the public health response to the COVID-19 pandemic [1].

The COVID-19 pandemic has also created a demand for timely data, leading to a surge in mobile phone surveys for tracking the impacts of and responses to the pandemic that have been conducted in many countries in Africa and Asia and in the United States [7-14]. The proliferation of mobile phone networks and mobile telephone affordability has contributed to the high penetration of mobile phone users and growth in mobile phone subscriptions [15]. The growth curve for the increase in mobile phone surveys did not match the exponential growth in mobile phone subscriptions until the COVID-19 pandemic.

A global survey of National Statistical Offices shows that over 80% are collecting data related to the COVID-19 pandemic; however, due to safety concerns, face-to-face survey data collection was suspended in the overwhelming majority of countries, and National Statistics Offices are relying on mobile phone surveys [7]. Many of these surveys aim to quantify the impacts of the COVID-19 pandemic as well as populations’ knowledge of COVID-19, engagement in preventive measures, and vaccine acceptance. Mobile phone surveys became the preferred mode of data collection across low- and middle-income countries (LMICs) when face-to-face data collection was paused due to physical distancing measures [14].

The impact of COVID-19 pandemic has varied throughout the country. In Sri Lanka, the first case of COVID-19 was detected in a traveler from China on January 27, 2020 [16]. The first locally acquired case of COVID-19 was confirmed 6 weeks later, on March 11, 2020 [17]. During the first wave, March 2020, the government of Sri Lanka acted swiftly to contain transmission, with very stringent public health measures, NPIs, and physical distancing, which included a complete island-wide lockdown, contact tracing and isolation, and quarantine of all inbound passengers [18]. The Sri Lankan government also closed public health clinics and started delivering routine health checks and medication directly to the homes of patients [19]. In addition to a strictly enforced early lockdown, relatively high testing rates and well-established health care and public health surveillance systems helped prevent many cases and deaths [19]. However, Sri Lanka experienced an increase of cases starting in April 2021 due to the Delta variant.

During the third wave of cases, in April 2021, the country moved from country-wide to area-based lockdowns determined by the high prevalence of cases. However, unlike the first wave, the postal service was not given access to high-risk areas, so they could not distribute medicine to these areas. The Sri Lankan government increased the bed capacity in quarantine and treatment centers and expanded the availability of intensive care unit beds in hospitals. The supply of test kits and personal protective equipment were streamlined to ensure a continuous supply of materials for hospitals. The government also procured vaccines, which arrived in April 2021. During our study period, Pfizer, Covishield, Sinopharm, and Sputnik vaccines were available to Sri Lankan residents. As of May 12, 2022, there were a total of 663,614 confirmed cases of COVID-19 in Sri Lanka with 16,510 deaths and 67.76% of the population was fully vaccinated [20].

In this study, we piloted 2 cross-sectional mobile phone surveys among Sri Lankan residents during the COVID-19 pandemic, specifically in May-June 2020 and April-May 2021. The primary objective was to test the feasibility of the mobile phone survey and to enable comparisons over time for specific questions. The first survey aimed to evaluate the use and effectiveness of a medicine delivery system for patients with noncommunicable disease (NCD) that was implemented during the initial stages of the pandemic. This system was designed to provide NCD medications to patients during the all-island curfew, which mandated residents to stay indoors for approximately 2 months as a measure to restrain the spread of COVID-19. Through this system, patients with NCD could have their medications delivered free of charge to their doorstep, either through their usual pharmacy or a government clinic. The medicine delivery system launched in March 2020 and remained operational throughout both survey periods. The second survey aimed to assess knowledge, attitudes, and practices related to practicing physical distancing and wearing a mask or facial covering. In addition, it aimed to understand access to and barriers of COVID-19 testing, measure acceptance of COVID-19 vaccines, and briefly reevaluate the use of the medicine delivery system among the adult Sri Lankan population. Both surveys were designed to capture data from the general population of mobile phone subscribers in Sri Lanka during the COVID-19 pandemic.

The objective of this study is to present a comprehensive summary of the findings derived from the pilot of 2 cross-sectional surveys conducted among adult Sri Lankan population during the COVID-19 pandemic. Furthermore, this study provides detailed insights into the methodology used in these surveys. The information presented herein will be invaluable for governments and decision makers seeking to undertake their own mobile phone surveys. In addition, these surveys contribute to the arsenal of e-surveillance tools and offer valuable insights into the feasibility of assessing nationally representative self-reported data for COVID-19.

## Methods

### Survey Design

Study data were collected and managed using Surveda, an open-source survey tool developed by the NCD Mobile Phone Survey Data 4 Health Initiative. Both Surveda and the Data 4 Health (D4H) initiative have been previously described elsewhere [21,22]. This activity was reviewed by Centers for Disease Control and Prevention (CDC) and was conducted in accordance with applicable federal law and CDC policy (eg, 45 C.F.R. part 46.102(l)(2), 21 C.F.R. part 56; 42 U.S.C. §241(d); 5 U.S.C. §552a; 44 U.S.C. §3501 et seq). The study protocol and procedures for the surveys were reviewed and approved by the Office of the Associate Director for Science within the Center for Global Health at the CDC under the D4H initiative. Approval was received for a nonresearch determination. In addition, the study protocol and procedures were reviewed and approved by the Ethics Review Committee at the Sri Lanka Medical Association. The questionnaire topics included the need for prescription medications during the pandemic, usage of the medication delivery system at government clinics and private pharmacies, and the assessment of the delivery system’s speed. The Sri Lankan Ministry of Health (MOH) developed unique questions and used questions that were previously validated in other recent COVID-19 research [23,24].

### Survey Settings

The surveys were conducted through automated, prerecorded messages, known as interactive voice response (IVR), sent to adult Sri Lankan people. The surveys were conducted in Sinhala, Tamil, and English. All respondents provided verbal consent. Consent was asked as the first question after language selection and included a description of the goals of the study and a reminder that respondents could refuse to answer any question and end the interview at any time. Respondents responded by choosing one of two prompts that corresponded with “yes” or “no.” Those who selected “no” and declined to participate were thanked and the call was ended. Consent was recorded and maintained as part of the final dataset.

Respondents were contacted between 8 AM and 8 PM, 7 days a week, with a maximum of 3 contact attempts made at 26-hour intervals. These contact attempts encompassed the initial contact to start the survey, any subsequent attempts to begin the survey, and recontacts if the survey was prematurely interrupted by the respondent due to reasons unrelated to interview completion (eg, the respondent hung up or experienced connectivity issues).

Reverse billing, which prevents respondents from being charged for airtime while responding to the survey, was set up with each mobile network operator in Sri Lanka and airtime costs were paid for by the D4H Initiative. Respondents were not provided with an incentive. After each completed interview, we provided respondents a toll-free number to call if they had questions about the survey and we reminded them to continue their medications for NCDs.

### Population and Sampling

According to census estimates, Sri Lanka had a population of over 22.9 million people in 2018, with 115.06 mobile phone subscriptions for every 100 people in Sri Lanka [25,26]. The sample design for both surveys was developed so that national-level population estimates could be generated. As a first step, sample sizes were calculated to produce estimates of acceptable precision for the overall population. The respondent sample size for overall estimates for both surveys was determined using the standard sample formula:



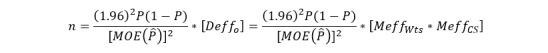



which takes into consideration the estimated prevalence of the risk factor (P), precision of the estimate desired margin of error (MOE(𝑃̂)), and overall design effect (Deff_O_.) The overall design effect is defined as the multiplicative increase in variance due to the cluster sample design (*MeffCS*) and the coefficient of variation among all sample weights (*MeffWts*). Considering this was a multi-risk survey, a prevalence of 50% was assumed in the determination of the sample size. The margin of error was assumed to be 5%, and the overall design effect was assumed as 1.25. The estimated sample size using the assumptions was calculated as:



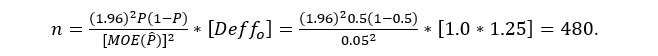



As a second step, to ensure that precision requirements were met for each sex, the sample size target in each sex was inflated by the proportion of the general population using the smaller population sex (males) as the reference. The ratio of the population distribution for males (46.3%) and females (53.7%) was calculated as 0.537÷0.463=1.16 females to 1 male. To inflate the sample size to produce estimates of acceptable precision by sex, 480 was used for males (480×1=480) and 556 for females (480×1.16=556). The total number of required interviews was calculated as 1036 (480+556) for the first survey.

The sample size for the second survey was calculated using the same formula used for the first survey. The targeted population for the second survey was adults aged 18 years and older. The assumptions on prevalence, margin of error, and overall design effect remained the same, namely prevalence of the risk factor=50%, precision of the estimate desired margin of error=5%, and Deff_o_=1.25. The ratio of the population distribution for males (48%) and females (52%) was calculated as 0.520÷0.480=1.10 females to 1 male. To inflate the sample size to produce estimates of acceptable precision by sex, 480 was used for males (480×1=480) and 528 (480×1.10=528). The total number of required interviews was calculated as 1008 (480+528). For both surveys, the required sample sizes were allocated proportionally to the mobile network market share in Sri Lanka (Dialog, Mobitel, Etisalat, Hutch, and Airtel).

The sample design for both surveys used a 2-phase sampling strategy. In the first phase, a random sample of mobile phone numbers (MPNs) from a frame of active subscribers, excluding those MPNs that were registered on national Do Not Call registries, was provided by a third-party company named Sample Solutions, Inc. In the second phase, respondents from the first phase were stratified into sex strata proportional to the general population.

### Participants and Data Collection Timeline

The eligibility criteria for the first survey were Sri Lankan residents aged 35 years and older with any NCD and access to a mobile phone. The age eligibility criterion for this survey was set, by recommendation of the MOH, based on our target population of adults with a need for NCD medicine. The eligibility criteria for the second survey were the Sri Lankan population aged 18 years and older with access to a mobile phone, with the target population being all adults living in Sri Lanka.

The first survey took place between May 7 and June 29, 2020, and the second survey took place between April 19 and May 9, 2021. The first survey required data collection pauses for 11 days, on May 9, May 15-22, and May 26-27, 2020, to facilitate channel upgrades with the mobile network operator providers.

### Statistical Analysis

Following data collection, the sample was adjusted to the 2019 United Nations Population Estimates [27] to produce population-based estimates by age and sex. Sample demographics defined weighting classes to ensure that the final weights summed to the specified population totals. These calibrated weights are the final adjusted sample weights that were used for analysis.

SAS (version 9.4; SAS Institute) and R Statistical Software (version 4.1.3; R Core Team 2022) were used for the analysis [28]. The analysis dataset included data from respondents with complete and partial data (answered a minimum of 6 questions). Data for any respondents who did not partially or fully complete an interview were excluded from the final dataset. Basic descriptive characteristics including frequencies for categorical variables and means and SDs for continuous variables were calculated. For binary and categorical response options, the percentage of respondents who selected each response was computed using the weighted data. For all proportions, 2-sided 95% CIs were calculated. To explore potential differences between sexes, second-order (Satterthwaite) Rao-Scott chi-square tests were conducted. To compare the responses regarding the use of the medicine delivery service between the first and second surveys, the second survey data was subset to include those aged ≥35 years and independent 2-sample *z* tests were conducted.

The overall response rate was the product of the phase 1 Response Rate (number of MPNs screened for eligibility [ie, provided age and sex] out of the number of MPNs dialed) and the phase 2 Response Rate (the proportion of interviews completed out of all interviews started in each stratum). The cooperation rate was calculated by dividing the number of completed interviews by the number of eligible respondents. The productivity of the sampling approach was determined by calculating the number of phone numbers dialed to yield a completed interview and an eligible respondent. The median duration for survey completion was computed using fully completed interviews only.

### Ethical Considerations

The study protocol and procedures for the surveys were reviewed and approved by the Office of the Associate Director for Science within the Center for Global Health at the Centers for Disease Control and Prevention under the Data for Health (D4H) initiative, receiving approval for a nonresearch determination. In addition, the study protocol and procedures were reviewed and approved (approval number NIHS/ERC/21/16) by the ethics review committee at the Sri Lanka Medical Association. Informed consent was obtained from all respondents, ensuring that participants were fully aware of the study’s purpose, procedures, and their right to opt out at any time. The data collected during the survey was anonymized or deidentified to protect the privacy and confidentiality of the respondents, safeguarding individual identities throughout the study. Respondents were not provided with any incentive for their participation in the survey.

## Results

### Respondent Characteristics

A total of 5001 adults aged 35 years or older completed the first survey (Survey 1 [T1]), while a total of 1250 adults aged 18 years or older completed the second survey (Survey 2 [T2]). In terms of sex distribution, the first survey consisted of 65% (3253/5001) male and 35% (1748/5001) female respondents, whereas the second survey had 49.6% (620/1250) male and 50.4% (630/1250) female respondents. Approximately half of the T1 respondents ((2622/5001, 52.4%) fell within the 35-44 years age range, while 54.4% (680/1250) of the T2 respondents were aged between 18 and 34 years. The majority of interviews were completed in Sinhala, accounting for 86.8% (4340/5001) in T1 and 87% (1087/1250) in T2. In terms of the sociodemographic structure, the first sample included a higher proportion of males and younger adults compared with the general population, while the second sample had a higher representation of younger adults. Refer to Table 1 for further details on respondent demographics.

**Table 1:** Characteristics of respondents in 2 cross-sectional mobile phone surveys on COVID-19 in Sri Lanka, 2020-2021.

Characteristics			T1^a^ (n=5001), n (%)		T2^b^ (n=1250), n (%)		Population^c^ (n=22.9 million), %
Interviews							
	Complete	4736 (94.7)		950 (76)		—^d^	
	Partial	265 (5.3)		300 (24)		—^d^	
Sex							
	Male	3253 (65)		620 (49.6)		52%	
	Female	1748 (35)		630 (50.4)		48%	
Age (years)							
	18-34	—^d,e^		680 (54.4)		33.2%	
	35-44	2622 (52.4)		289 (23.1)		20.5%	
	45-54	1515 (30.3)		184 (14.7)		17%	
	55 and older	864 (17.3)		97 (7.8)		29.3%	
Language							
	Sinhala	4340 (86.8)		1087 (87)		87%^f^	
	Tamil	449 (9)		134 (10.7)		28.5%^f^	
	English	212 (4.2)		29 (2.3)		23.8%^f^	

^a^T1: Survey 1.

^b^T2: Survey 2.

^c^Information obtained from and Sri Lanka Census of Population and Housing 2018 Projections and The World Factbook [25].

^d^Not applicable.

^e^The eligibility criteria for this survey was ≥35 years of age.

^f^Sums to more than 100% because some respondents gave more than one answer on the census.

### Survey Metrics, Call Outcomes, and Response Rates

For T1, the median duration of completed interviews was 5.4 (IQR 4.3-6.2) minutes. For T2, the median duration of completed interviews was 7.9 (IQR 7.3-8.8) minutes. Among those who fully completed the interview, Surveda dialed each respondent on average 1.4 times in T1 and 1.5 times in T2.

**Figure 1:**
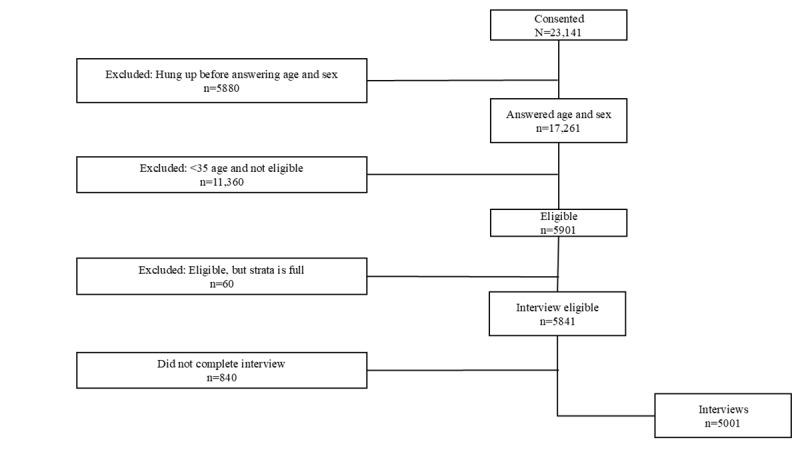
Survey 1 flowchart.

For T1, Surveda dialed 198,152 MPNs. Of these, 23,141 consented and 17,261 provided the age and sex information necessary to be eligible to participate. This is a productivity rate of 34 MPNs needed to yield an eligible contact. Of the 17,261 respondents, 11,360 were ineligible (younger than 35 years) and 60 respondents of eligible age were rejected due to stratum sample size being full. The result was 5841 eligible respondents, of which 5001 provided complete or partial interviews. For the first survey, the majority (4736/5001, 94.7%) of respondents completed the interview and only 5.3% (265/5001) provided a partial interview. This is a productivity rate of 40 MPNs needed to yield a completed interview. The number of interviews collected exceeded the sample size calculation, given the need to evaluate responses from those who had used the medicine delivery service. The overall response rate was 7.5%. The completion rate was 85.6% (5001/5841). The flow chart for respondents once consented is shown in Figure 1.

**Figure 2:**
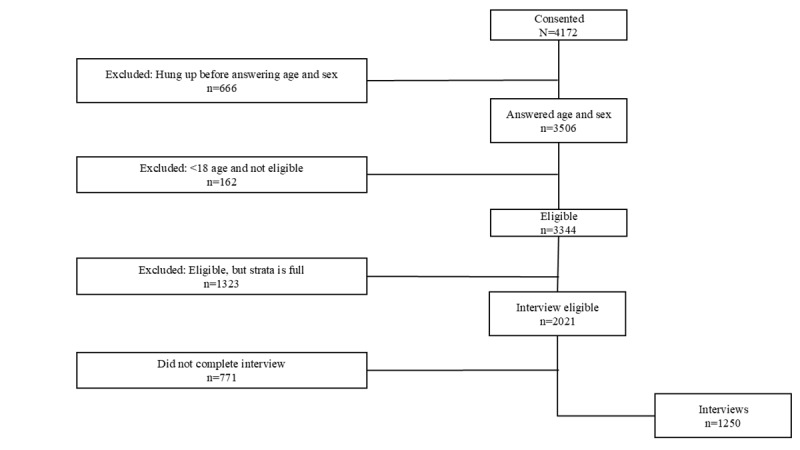
Survey 2 flowchart.

For T2, Surveda dialed 19,233 MPNs. Of these, 4172 consented and 3506 provided the age and sex information necessary to be eligible to participate. This is a productivity rate of 6 MPNs needed to yield an eligible contact. Of these 3506, 162 were ineligible (less than 18 years old), and 1323 respondents of eligible age were rejected due to stratum sample size being full. The result was 2021 eligible respondents, of which 1250 provided complete or partial interviews. For the second survey, more than two-thirds (950/1250, 76%) fully completed the interview and 24% (300/1250) partially completed the interview. This is a productivity rate of 15 MPNs needed to yield a completed interview. The overall response rate was 10.9%. The completion rate was 61.9% (1250/2021). The flow chart for respondents once consented is shown in Figure 2. Table 2 shows the call outcomes, or dispositions, for both surveys.

**Table 2:** Final disposition codes for all dialed phone numbers in 2 mobile phone surveys on COVID-19 in Sri Lanka, 2020-2021.

Disposition	Definition	T1^a^, n (%)	T2^b^, n (%)
Complete	Answered all survey questions	4736 (2.4)	950 (4.9)
Partial	Answered at least one question COVID-19–related question in T1 and at least one topic area (minimum of 6 questions) in T2 but did not finish the survey	265 (0.1)	300 (1.6)
Breakoff: eligible	Answered age and sex questions but did not answer any COVID-19–related questions	840 (0.4)	933 (4.9)
Ineligible: age	Younger than 35 years in T1 and younger than 18 years in T2	11,360 (5.7)	162 (0.8)
Ineligible: quotas	Age-sex quotas were full	60 (0)	1323 (6.9)
Refused	Refused consent	7522 (3.8)	5388 (28)
Unknown eligibility	Answered some questions but stopped before completing eligibility	5880 (3)	504 (2.6)
No answer	No answer, possibly nonworking number	167,489 (84.5)	9673 (50.3)
Total	—^c^	198,152	19,233

^a^T1: Survey 1.

^b^T2: Survey 2.

^c^Not applicable.

### Nonpharmaceutical Interventions, COVID-19 Testing, Diagnosis, and Treatment, and Vaccine Acceptance in the T2 Survey

In general, adult Sri Lankan persons adhered to NPI COVID-19 mitigation strategies implemented by the MOH. In total, 3 out of 4 adults reported that they avoided public places (888/1175, 75.6%); over two-thirds avoided public transportation (808/1173, 68.9%); and 9 out of 10 practiced physical distancing (1046/1167, 89.7%). Approximately, 9 out of 10 adult Sri Lankan residents reported always wearing a face mask when they were out in public (976/1071, 92.4%), and always covering their nose and mouth while masking in the previous seven days (976/1041, 93.8%). Table 3 shows results of the NPI-related questions.

Approximately 1 out of 10 Sri Lankan population reported feeling sick in the last 7 days and being tested for COVID-19; of those, the majority received a polymerase chain reaction test (112/161, 70%). Among those tested, 8.6% (13/160) reported being diagnosed with COVID-19. A majority (7/13/, 60.5%) of those diagnosed, reported that they received treatment for COVID-19. More males than females reported being tested for COVID-19 (98/554, 17.8% vs 61/578, 10.6%, respectively), and this difference was statistically significant (*P*<.005). Among adult Sri Lankan residents who were not tested, 4.9% (46/959) reported that they tried to get a COVID-19 test but were not able to do so. The most common reason for not getting a test was lack of availability (19/41, 46.5%). Table 3 shows results of testing, diagnosis, and treatment questions.

Among those who answered the question on whether they would get a COVID-19 vaccination if it was available today, the majority reported that they definitely or probably would get the vaccination (781/1190, 65.7%). Males and females were equally as likely to take the vaccination. Most would prefer to receive the vaccination from a government hospital (636/729, 87.3%). Among those who probably or definitely would not get the vaccination (407/1190, 34.3%), approximately a third were deciding to wait (152/413, 36.8%), 30.7% (126/413) thought it was not safe, 13.4% (55/413) did not believe in vaccination, 9.8% (40/413) did not think it was effective, and 9.4% (38/413) did not think they would get sick with COVID-19. Males and females reported similar reasons for not wanting to get vaccinated; however, more men than women thought that the vaccine was not safe (74/208, 35.7% vs 52/205, 25.8%, respectively). Table 3 shows results of vaccine acceptance questions.

**Table 3:** Nonpharmaceutical interventions, COVID-19 testing, diagnosis and treatment and vaccine acceptance in a mobile phone survey on COVID-19 in Sri Lanka, 2021 (T2).

					Overall						Males						Females						*P* value	
					Value, n (%)			95% CI			Value, n (%)			95% CI			Value, n (%)			95% CI				
Nonpharmaceutical interventions																								
	Avoided public places			1175 (75.6)			72.9-78.4			588 (76.2)			72.4-80.0			587 (75.1)			71.1-79.1			.71		
	Avoided public transportation			1173 (68.9)			66.0-71.9			585 (68.3)			64.1-72.4			588 (69.6)			65.3-73.8			.67		
	Avoided social contact			1167 (89.7)			87.7-91.6			584 (89)			86.3-91.7			583 (90.2)			87.5-93.0			.53		
	Always wore face mask in public (in the last 7 days)			1071 (92.4)			90.6-94.1			533 (91.9)			89.4-94.4			538 (92.8)			90.3-95.3			—^a,b^		
	Always covered nose and mouth with the face mask			1041 (93.8)			92.2-95.4			519 (94)			91.9-96.2			522 (93.6)			91.2-96.0			.78		
COVID-19 testing, diagnosis, and treatment																								
	Among adult Sri Lankan residents^c^																							
		COVID-19 diagnosis	1126 (1.2)			0.5-1.9			553 (1.7)			0.6-2.9			573 (0.7)			0.0-1.5			.14			
		Treatment for COVID-19	979 (0.8)			0.2-1.4			465 (1.4)			0.3-2.5			514 (0.3)			0.0-0.8			.09			
	Among adult Sri Lankan residents who answered “yes” to feeling sick in the previous 7 days^d^																							
		Tried to get a test for COVID-19	959 (4.9)			3.4-6.5			452 (6.1)			3.7-8.5			507 (3.9)			1.9-6.0			.17			
		Tried to get a test but it was not available	41 (46.5)			29.2-63.7			26 (54.9)			34.0-75.8			15 (35.1)			7.3-62.9			.51			
		Tried to get a test, but I was not eligible for testing	41 (28.4)			13.5-43.3			26 (21.2)			4.4-38.0			15 (38.3)			11.6-64.9			.51			
		Tried to get a test, but it was too expensive	41 (25.1)			9.8-40.4			26 (23.9)			6.6-41.2			15 (26.7)			0.0-53.9			.51			
	Tested for COVID-19			1132 (14)			11.8-16.2			554 (17.8)			14.3-21.2			578 (10.6)			7.9-13.4			.002^e^		
		Polymerase chain reaction test	161 (70)			62.2-77.9			98 (69.5)			59.4-79.7			63 (70.8)			58.5-83.2			.98			
		Rapid Antigen Test	161 (16.1)			9.8-22.3			98 (16.8)			8.6-25.1			63 (14.9)			5.4-24.5			.98			
		Both	161 (9.8)			5.0-14.6			98 (9.9)			3.5-16.3			63 (9.6)			2.6-16.6			.98			
		Did not know test type	161 (4.1)			0.0-8.2			98 (3.7)			0.0-8.8			63 (4.6)			0.0-11.6			.98			
	COVID-19 diagnosis (among those tested)			160 (8.6)			3.7-13.6			98 (9.9)			3.5-16.3			62 (6.6)			0.0-14.4			.53		
	Treatment for COVID-19 (among those diagnosed)			13 (60.5)			29.5-91.5			10 (66.6)			33.3-99.9			3 (45.9)			0.0-100.0			.33		
Vaccine acceptance																								
	Would get a COVID-19 vaccination if it was available today																							
		Definitely	1190 (42.2)			39.1-45.4			589 (44.4)			40.0-48.8			601 (40.2)			35.7-44.7			.30			
		Probably would	1190 (23.5)			20.9-26.2			589 (21)			17.5-24.5			601 (25.8)			21.9-29.8			.30			
		Probably would not	1190 (26)			23.2-28.7			589 (26)			22.2-29.8			601 (25.9)			22.0-29.8			.30			
		Definitely would not	1190 (8.3)			6.7-10.0			589 (8.6)			6.3-11.0			601 (8)			5.7-10.3			.30			
	Where would you want to receive your vaccination																							
		Government hospital	729 (87.3)			84.6-90.1			366 (90.1)			86.7-93.4			363 (84.8)			80.5-89.1			.06			
		Private hospital	729 (12.7)			9.9-15.4			366 (9.9)			6.6-13.3			363 (15.2)			10.9-19.5			.06			
	Reason for not wanting the vaccine																							
		Waiting to decide	413 (36.8)			31.5-42.0			208 (35)			28.0-42.1			205 (38.4)			30.7-46.1			.32			
		Do not think it is safe	413 (30.7)			25.8-35.5			208 (35.7)			28.6-42.8			205 (25.8)			19.3-32.3			.32			
		Do not believe in vaccination	413 (13.4)			9.9-16.9			208 (10.6)			6.7-14.6			205 (16)			10.4-21.7			.32			
		Do not think it is effective	413 (9.8)			6.6-13.1			208 (9.7)			5.1-14.3			205 (10)			5.5-14.5			.32			
		Do not think you will get COVID-19	413 (9.4)			5.9-12.8			208 (9)			4.3-13.6			205 (9.7)			4.7-14.7			.32			

^a^Not applicable.

^b^Test could not be completed because at least one cell had 0.

^c^These estimates are among the survey sample, regardless of whether the respondent reported feeling sick in the previous 7 days.

^d^Only those respondents who answered “yes” to feeling sick in the last 7 days were asked about testing, diagnosis, and treatment. These estimates are among only those respondents who reported feeling sick in the last 7 days.

^e^Significance *P*<.005.

### Medicine Delivery Service Knowledge and Use Comparison Between T1 and T2

In total, 3 questions were asked in both T1 and T2, allowing for a direct comparison. When comparing the results of T1 and T2, we observed that a higher proportion of respondents reported needing medicine in T1 compared with T2. In addition, a greater percentage of respondents in T1 (617/755, 81.8%) were aware of the medicine delivery service compared with T2 (266/365, 72.9%). Furthermore, among those who required medicine, a larger proportion of respondents reported using the medicine delivery service in T1 than in T2 (427/552, 77.4% vs 93/177, 52.9.%). For further details on the comparison of respondents’ needs, knowledge, and use of the medicine delivery service between T1 and T2, refer to Table 4.

**Table 4:** Respondents’ needs, knowledge, and use of medicine delivery service across time in mobile phone surveys on COVID-19 in Sri Lanka, 2020-2021.

Survey item	T1^a^ (2020)		T2^b^ (2021)		Absolute difference	*P* value
	Value, n (%)	95% CI	Value, n (%)	95% CI		
Needed medicine	1040 (24)	22.3-25.8	66 (13.5)	10.3-16.7	10.5	<.001
Knew of the medicine delivery service	755 (81.8)	78.1-85.5	365 (72.9)	68.7-77.0	8.9	<.001
Used the medicine delivery service	552 (77.4)	73.3-81.5	177 (52.9)	47.3-58.6	24.5	<.001

^a^T1: Survey 1.

^b^T2: Survey 2.

## Discussion

### Principal Findings

The COVID-19 pandemic has created urgent demand for timely data, leading to a surge in mobile phone surveys for tracking the impacts of and responses to the pandemic. In fact, there is evidence that the surge in mobile phone surveys during the COVID-19 pandemic may transition into a preferred mode of data collection in LMICs [14]. A key contribution of our study relative to the existing literature is its focus on repeated surveys. The MOH in Sri Lanka conducted 2 mobile phone surveys, 1 during 2020 and 1 during 2021. The data were collected quickly (44 days for T1 and 19 days for T2). These data add to the growing research base documenting that mobile phone surveys in countries with high mobile phone penetration, like Sri Lanka, are feasible and fast.

This research adds to the existing literature on response rates and completion rates for mobile phone surveys in LMICs. The overall response rates for our surveys were 7.5% and 10.9%. Previous research has shown that random digit dial IVR survey response rates are typically 8%-31% and are higher for those surveys where an incentive was provided [29,30]. The completion rates for our surveys were 85.6% for T1 and 61.9% for T2. Previous research for IVR mobile phone surveys have found a wide range of completion rates (23%-75%) [30]. Our findings indicate that our response rates and completion rates are consistent with previous research. Furthermore, our findings indicate that once a respondent was deemed eligible for survey response, they were more likely to complete the survey.

In seeking to limit the number of new infections of COVID-19, governments around the world have implemented guidelines about safe behaviors. We found high adherence to the NPIs mitigation strategies that were encouraged by the MOH during the COVID-19 response. For example, most respondents reported avoiding public places (888/1175, 75.6%), public transportation (808/1173, 68.9%), and practicing physical distancing (1046/1167, 89.7%). This finding is higher than a study conducted in Ethiopia where 40.6% reported avoiding public spaces [31]. Our findings are consistent with other countries in Asia, such as Malaysia, where 83.4% reported avoiding public spaces [32]. This survey found that adherence of Sri Lankan population toward wearing a facemask as mitigation measure was high; most Sri Lankan people reported always wearing a face mask when they were out in public (976/1071, 92.4%) and most reported that they always properly wear the mask by covering their nose and mouth (976/1041, 93.8%). This is higher than studies conducted in Egypt (57%) [33], the United States (77%) [34], and India (89.5%) [35].

Once the initial lockdown was eased in Sri Lanka, the COVID-19 response consisted of surveillance, contract tracing, and testing as the core components [36]. The systematic and extensive contract tracing program was launched using the existing public health system and collaborating with security forces and the national intelligence service [37]. The surveillance and testing strategy used by Sri Lanka was based on the principle that early case detection and diagnosis by laboratory confirmation is critical for the prevention of community disease transmission [38]. Regarding testing for COVID-19, we found that among adult Sri Lankan persons who were not tested, 4.9% (46/959) reported that they tried to get a COVID-19 test but were not able to do so. The finding that so few respondents wanted a test but could not get one suggests that most of the demand for tests was being met and that the government’s strategy of surveillance and contract tracing was working as intended.

Widespread acceptance of COVID-19 vaccines is crucial for achieving sufficient immunization coverage to end the COVID-19 pandemic. This research adds to the scientific literature investigating vaccination attitudes in LMICs and in Sri Lanka specifically. While the Government of Sri Lanka commenced its COVID-19 vaccination program on January 28, 2021, the vaccine was not yet widely available at the time of the T2 survey. We found that approximately 66% (781/1190) of Sri Lankan residents reported that they definitely or probably would get the vaccination if it was available today. In contrast, vaccine acceptance in other LMICs has been shown to be around 80% [12]. Furthermore, a study using a web-based self-administered questionnaire conducted in January-March 2021 found that 92.4% of Sri Lankan population were likely or extremely likely to get vaccinated against COVID-19 [39].

The possible explanation for the lower vaccine acceptance may be because of misinformation surrounding the vaccine or a preference for a particular brand of vaccine [39]. Published research carried out largely in high-income countries cites concerns about the safety of COVID-19 vaccines, including the rapid pace of vaccine development, as one of the primary reasons for hesitancy, but data from LMICs have been limited. Deciding to wait and concerns about safety were reported as the drivers of vaccine hesitancy in Sri Lankan people. The current survey contributes to the emerging picture of global vaccine acceptance by asking questions about vaccine acceptance and reasons for hesitancy. In addition, the survey findings around vaccine acceptance (66%) suggests a need for increased public health campaigns on the safety and effectiveness of the vaccine.

When assessing the medicine delivery system set up by the MOH to provide a continuous supply of NCD medicine during the pandemic, we found that more respondents knew about the service and used the service in 2020 compared with 2021. The possible explanation for decreased knowledge and use might be that during 2021 there were less restrictions than in 2020 and Sri Lankan population could leave their homes to obtain NCD medicine at their usual clinic or pharmacy rather than use the medicine delivery service. In addition, there may have been more public awareness during the rollout of the medicine delivery service. However, our survey found that Sri Lankan residents (n=177) reported using the service in 2021, which caused the MOH to continue to offer the program.

The MOH has presented the survey findings from the first survey to high-level officials and used the survey data to inform care practices. The MOH set up temporary mobile NCD clinics in high-risk areas that are inaccessible by postal delivery, set up permanent guidelines on frequency of issuing medicines to increase supply provided to patients, and set up a temporary system for pharmacies to receive and provide medicine to patients. The MOH also used the data from both surveys to inform health communication messaging.

### Limitations

The results of these mobile phone surveys are derived from responses provided by mobile phone owners, potentially introducing selection bias that could favor respondents with higher education or socioeconomic status. However, it is worth noting that mobile phone penetration is high in Sri Lanka, with 115.06 mobile phone subscriptions for every 100 individuals. Nevertheless, it is important to acknowledge that previous research has indicated that mobile phone surveys using mobile internet technology may exhibit bias against individuals with lower socioeconomic status, as smartphones are typically concentrated among the affluent population in urban areas, and coverage of communication networks used by smartphones remains limited in LMICs [29]; Therefore, we chose to use a mobile phone–based survey that does not rely on mobile internet technology (ie, IVR) to mitigate this potential bias.

High mobile phone ownership helps reduce coverage bias, which refers to how well a sample represents the larger population. However, it is important to note that women are considerably less likely than men to own a mobile phone both globally and in Sri Lanka [40]. Further research is needed to explore effective methods for better engaging women and rural residents in mobile phone surveys, such as using motivational call greetings, calling at specific times, implementing survey prenotifications, and providing incentives [41,42]. Nonetheless, adjusting data from mobile phone samples, as we have done, particularly by sex and age, has been shown to reduce noncoverage bias by yielding demographic characteristics [41] and health indicators that are comparable to those obtained from household samples.

Another limitation of this study is its retrospective nature, which may affect the relevance of the findings to the current context, particularly in relation to evolving qualitative characteristics such as vaccine acceptance and adherence to NPIs. While historical data offers valuable insights into behavioral trends, the attitudes and behaviors observed during the study period may differ significantly if the survey were conducted more recently. In addition, although the study’s findings provide useful information for future pandemic preparedness in Sri Lanka, the context-dependent nature of these insights should be acknowledged. While the mobile phone survey methodology has the potential to be applied to other public health challenges, we recognize that transferring findings from one context to another may require specific adaptations. Therefore, caution should be exercised when generalizing these results to different public health scenarios, and further research may be needed to validate their applicability.

Finally, it is important to acknowledge that the data collected in this study relied on self-reporting, and respondents may be less inclined to disclose socially or culturally undesirable information, potentially leading to an overestimation of adherence to mitigation strategies and vaccine acceptance. Nevertheless, it is worth noting that the anonymous nature of data collection in this study helps mitigate the risk of social desirability bias.

### Conclusion

These repeated cross-sectional mobile phone surveys provided a rapid method for collecting COVID-19–related data during the pandemic, enabling timely information to be delivered to the Sri Lankan MOH. The surveys revealed that, overall, adult Sri Lankan persons reported adherence to the NPI COVID-19 mitigation strategies implemented by the MOH. Furthermore, approximately 66% of Sri Lankan population expressed a willingness to receive the COVID-19 vaccination if it were available today, highlighting the potential for targeted health information campaigns. Notably, the high usage of the medicine delivery system prompted the government to continue offering the service. The survey findings were used by the MOH in real time to inform care practices, such as increasing the supply of medicine provided to patients, and shape health communication messaging. These data contribute to the growing body of literature on mobile phone surveys in LMICs. This study underscores the use of mobile phone surveys as a rapid means of filling data gaps when face-to-face data collection is impractical. The adoption of repeated cross-sectional mobile phone surveys facilitates real-time data collection and use, providing a valuable tool for evidence-based programmatic and policy decision-making, particularly given the evolving nature of the COVID-19 epidemiology and understanding.

## Abbreviations

CDC: Centers for Disease Control and Prevention
D4H: Data 4 Health
IVR: interactive voice response
LMIC: low- and middle-income country
MOH: Ministry of Health
MPN: mobile phone number
NCD: noncommunicable disease
NPI: nonpharmaceutical intervention
T1: Survey 1
T2: Survey 2

## Data Availability

The datasets generated during and/or analyzed during this study are available in the ncdmobile website repository [43].
